# Quantifying the Endogeneity in Online Donations

**DOI:** 10.3390/e23121667

**Published:** 2021-12-11

**Authors:** Peng Wang, Jinyi Li, Yinjie Ma, Zhiqiang Jiang

**Affiliations:** 1 School of Business, East China University of Science and Technology, Shanghai 200237, China; pwang@mail.ecust.edu.cn (P.W.); yjma@mail.ecust.edu.cn (Y.M.); 2 Research Center for Econophysics, East China University of Science and Technology, Shanghai 200237, China; 3 Department of Mathematics, Eberly College of Science, Penn State University, State College, PA 16802, USA; cassieli1310@gmail.com

**Keywords:** charitable crowdfunding, endogeneity, branching ratio, Hawkes process, online donation

## Abstract

Charitable crowdfunding provides a new channel for people and families suffering from unforeseen events, such as accidents, severe illness, and so on, to seek help from the public. Thus, finding the key determinants which drive the fundraising process of crowdfunding campaigns is of great importance, especially for those suffering. With a unique data set containing 210,907 crowdfunding projects covering a period from October 2015 to June 2020, from a famous charitable crowdfunding platform, specifically Qingsong Chou, we will reveal how many online donations are due to endogeneity, referring to the positive feedback process of attracting more people to donate through broadcasting campaigns in social networks by donors. For this aim, we calibrate three different Hawkes processes to the event data of online donations for each crowdfunding campaign on each day, which allows us to estimate the branching ratio, a measure of endogeneity. It is found that the online fundraising process works in a sub-critical state and nearly 70–90% of the online donations are endogenous. Furthermore, even though the fundraising amount, number of donations, and number of donors decrease rapidly after the crowdfunding project is created, the measure of endogeneity remains stable during the entire lifetime of crowdfunding projects. Our results not only deepen our understanding of online fundraising dynamics but also provide a quantitative framework to disentangle the endogenous and exogenous dynamics in complex systems.

## 1. Introduction

Fundraising is a process of seeking and gathering money by engaging individuals, businesses, charitable foundations, or governmental agencies (https://en.wikipedia.org/wiki/Fundraising, accessed on 19 November 2021). As a new form of fundraising, crowdfunding takes advantage of the internet to collect funds through small contributions from a large number of contributors for commercial and charitable purposes. Commercial crowdfunding, including reward-based crowdfunding (e.g., Kickstarter and Indiegogo) and equity-based crowdfunding (e.g., SeedInvest), is mainly employed to support the venture financing of innovative businesses. Charitable crowdfunding, also called donation-based crowdfunding, helps individuals or non-profitable organizations collect money for themselves, friends, families, and even strangers who need assistance in urgent times. In charitable crowdfunding, the backers are mainly driven by altruistic motivations. As a promising form of charity, charitable crowdfunding has received considerable contributors and donations. In 2018, a survey on crowdfunding in America (https://nonprofitssource.com/online-giving-statistics/, accessed on 19 November 2021) revealed that almost 41% of respondents had made donations. In the meantime, in China, 20 charitable crowdfunding platforms registered in the Chinese Ministry of Civil Affairs had raised more than CNY 3.17 billion, which increased about by 30% in comparison to the donations in 2017 (http://www.charityalliance.org.cn/givingchina/12781.jhtml, accessed on 19 November 2021). Although charitable crowdfunding has attracted a great deal of attention in industry, the dynamics of the fundraising process still experience a lack of investigation.

The investigation of crowdfunding fundraising mainly focuses on how the sophisticated tangles of exogenous shocks and self-organizing evolution influence the dynamics of money collection [1,2,3]. Specifically, each crowdfunding project is accompanied by several seed donors in the initial phase and seed donors spread the campaign through their social networks to attract more kind people to donate and share, which may lead to the propagation of the campaign on social networks. Thus, these seed donors can be viewed as “innovators” [4] or “immigrants” [5], who introduce external resources or excite kind-hearted individuals to promote the charitable fundraising project. Usually, strong exogenous effects will trigger unexpected concerns. For example, an unexpected earthquake [6,7], a disastrous tsunami [8], and a sudden malady [9,10] naturally attract an inordinate amount of attention. Thus, early access to a great amount of “innovators” or “immigrants” will promisingly make crowdfunding projects gain a wider range of popularity, leading to bursts of donations in the early stage [2].

Due to the spreading of crowdfunding campaigns triggered by seed donors, more and more individuals are aware of the detailed information of campaigns, which, in turn, brings in more donations. This can be considered as the endogeneity in online donations, meaning the endogenous dynamics of attracting more people to donate through the donating and sharing of actions made by donors. On one hand, due to the peer similarity [11] and preferential attachment [12] in social networks, the internal exposure and cumulative prevalence of crowdfunding campaigns lead to positive feedback, which makes more and more people become involved in the cascading dynamics. On the other hand, similar to the fading novelty of an academic publication [12,13] and diminishing activity of a microblog [14], crowdfunding projects also exhibit a time decay pattern which deteriorates public attention. This generates an antagonistic effect against the positive feedback of internal exposure and cumulative prevalence. These two effects compete with each other and directly determine the rise and fall of the donating process. For those who are desperately struggling and seeking negligible assistance from acquaintances and strangers through the crowdfunding system, it is of great importance to investigate how exogeneity and endogeneity affect the performance of crowdfunding. Considering the donation process is non-Poissonian [15], quantifying the endogenous and exogenous components of charitable crowdfunding campaigns and modeling the corresponding diffusion process are key ingredients to boost fundraising, which provides a new channel for seriously sick people who cannot afford medical expenses to seek aid.

Recently, the Hawkes process has been widely applied to model the underlying cascading dynamics in many complex systems, such as earthquakes and aftershocks [16,17], price changes in financial markets [5,17,18,19,20], social sharing services [21], and disease dissemination [9], to list a few. By calibrating the Hawkes process to the empirical data, the occurring events can be decomposed into exogenous parts generated by external information and endogenous parts triggered by historical events. Our research interest here is to uncover how many donations are from the endogeneity, wherein donors attract more donors through their donating and sharing actions. The contributions of our paper are as follows. First, we extend the Hawkes processes to investigate the online donating processes of crowdfunding campaigns and especially pay attention to the self-excited effects in online donations, wherein donors attract potential donors. Our results complement the analysis of the donating events by means of the recurrence interval analysis [15]. Second, differing from the studies on uncovering the endogeneity in price changes in financial markets [5,18,19,22,23] and in digital currency markets [24], our work focuses on the endogeneity in the donations of online crowdfunding projects, which still experiences a lack of investigation. Our data set contains 210,907 projects, spanning over a period from October 2015 to June 2020, which allows us to reveal the dominating driving force in online donations. By calibrating to three Hawkes processes, we found that about 70–90% of the donations are endogenous, meaning that cascading the campaigns in social networks plays an important role in attracting donations. Third, it is argued that the level of endogeneity increases from 30% in 1998 to 70% in 2010 in financial markets because of algorithm trading [5,19]. However, how the level of endogeneity in online donations evolves with respect to the elapsing time is not clear. We thus fill this gap by calibrating the Hawkes processes to the online donations on different elapsing days. We surprisingly found that the level of endogeneity, measured by the branching ratio, is approximately a constant during the entire life of crowdfunding campaigns, indicating that there is a universal underlying law governing online donating processes.

This paper is organized as follows. Section 2 presents the data description. Section 3 briefly introduces the Hawkes processes and methods of goodness-of-fits. The results are given in Section 4. Section 5 provides a conclusion.

## 2. Data

Our data were retrieved from a famous medical crowdfunding platform in China, namely Qingsong Chou. A patient who lacks medical expenses can initiate a crowdfunding campaign to receive donations from the public. The campaign initiator receives online donations from the goodness and generosity of people through cascading his crowdfunding campaign in social networks. Obviously, the fundraising strongly depends on the speed and wideness of the campaign spreading. For each online donation, we retrieved the information of the donor, donating time, and donated amount.

**Figure 1:**
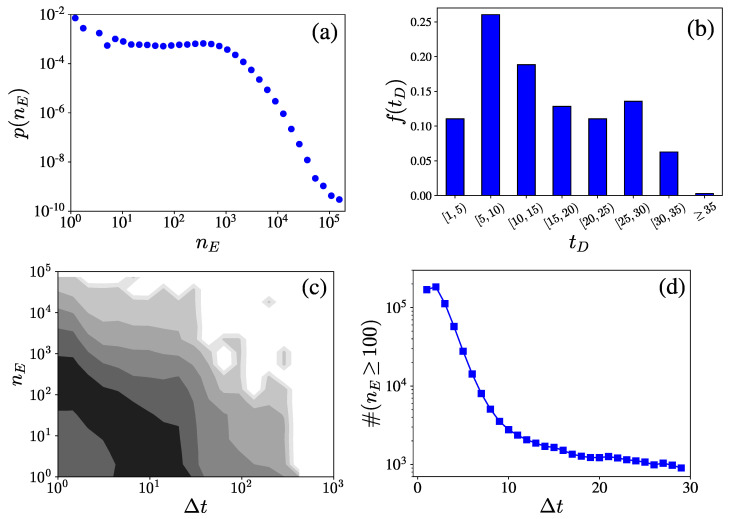
Statistical overview of data sets. (**a**) Probability distribution of campaign donating counts $n_{E}$. (**b**) Frequency of campaign fundraising days $t_{D}$. (**c**) Contour plots of the daily donating counts with respect to the elapsing days Δt. (**d**) Plots of the number of crowdfunding campaigns with more than 100 donating counts $\#(n_{E}>100)$ with respect to the elapsing days Δt.

We examined 210,907 projects spanning over a period from October 2015 to June 2020, from the Qingsong Chou platform and performed a preliminary analysis on the set of projects. Figure 1a illustrates the probability distribution of campaign donating counts $n_{E}$. One can observe that most of the crowdfunding campaigns receive less than one thousand donations. The frequency of fundraising days is shown in Figure 1b. As the default setting of the fundraising days is 30, nearly 95% of the campaigns raise money within one month. Usually, the patients urgently need to pay their medical expenses and are allowed to both stop money collections and withdraw funds at any time. Thus, one can see that more than 50% of the campaigns take less than two weeks to raise funds. We also illustrate the contour plots of donating counts $n_{E}$ with respect to the elapsing days Δt in Figure 1c. It is observed that the darkest belt nearly exhibits a remarkable power-law behavior, wherein $n_{E}$ decays from $\left[10^{2},10^{3}\right]$ to less than 10 within ten days. We further counted the number of projects whose donating counts were greater than 100 on each elapsing day and the corresponding number of projects $\#(n_{E}>100)$ are plotted with respect to the elapsing days Δt in Figure 1d. We found that $\#(n_{E}>100)$ sharply decreases with the increment of elapsing days. This indicates that the underlying diffusing process of crowdfunding projects usually dies out within several days. Thus, we only concentrated on the daily donating activities containing more than 100 events for a given crowdfunding project in the following analysis, as this ensures the obtaining of a reliable calibration [5].

## 3. Model

### 3.1. Hawkes Process

As crowdfunding campaigns spread on social networks, donations can trigger new donations. This is reminiscent of the self-excited Hawkes process, which is formulized as follows.

(1) $$\lambda \left(t\right)=\mu \left(t\right)+\sum_{t_{i}<t}h\left(t-t_{i}\right),$$

where λ(t) describes the conditional intensity of the fundraising process of crowdfunding campaigns, which also reflects the expectation of the number of donations within [t,t+dt]. μ(t) is the background intensity capturing exogenous donations, $h(t-t_{i})$ is the memory kernel function which describes the endogenous donations generated by past donations, and $t_{i}$ is the occurring time of the *i*-th donation. Obviously, the self-excited Hawkes process can be seen as a linear combination of exogenous and endogenous components, in which exogenous events generate daughters and daughters in turn generate daughters. This is equivalent to branching processes. As we know, the key parameter of the branching process is the branching ratio *n*, meaning the average number of daughters per mother. Filimonov and Sornette also use the branching ratio to measure the endogeneity of market dynamics [5]. The branching ratio *n* can be simply estimated via its definition.

(2) $$n=\int_{0}^{\infty }h\left(t\right)dt.$$

The branching process can be classified into three regimes according to the values of the branching ratio: (1) sub-critical (n<1), (2) critical (n=1), and (3) supercritical (n>1). Obviously, crowdfunding campaigns are expected to operate in a critical or supercritical regime, wherein one exogenous donation can bring in many subsequent endogenous donations. If the donating process is sub-critical, the fundraiser should broadcast his project as widely as possible to trigger exogenous donations.

Usually, the background intensity μ(t)=ω in the Hawkes process is assumed to be a constant and the memory kernel function h(t) takes the form of both an exponential function [5,19,24] and power-law function [16,20,24]. The exponential kernel indicates that the influence of the history donation exponentially decays with respect to the time elapsed since it occurred [25].

(3) $$h\left(t\right)=n\beta e^{-\beta t},$$

where *n* is nothing but the branching ratio and β describes how fast the past influence decays. The power-law kernel is proposed to capture the long memory in earthquake occurrence, which can be rewritten as

(4) $$h\left(t\right)=n\frac{\alpha c^{\alpha }}{{\left(t+c\right)}^{1+\alpha }}$$

where *n* is the branching ratio, α is the decay parameter, and *c* is the regularization parameter that assures the integrability of the power-law kernel. As the likelihood function of the Hawkes process can be derived theoretically, we were also able to determine the parameters of the Hawkes process through the maximum likelihood estimation (MLE) [26,27].

### 3.2. Renewal Hawkes Process

Recently, a generalized Hawkes process, called the renewal Hawkes (RHawkes) process, was proposed, in which the arrival of immigrant (exogenous) events is modeled by a more flexible renewal process rather than a fixed Poisson process [28]. The background intensity μ(t) in Equation (1) is not a constant anymore but varies as a function of time. We can model the renewal process by simply assuming that the waiting time between the immigrant events follows a Weibull distribution. Thus, the associated background intensity can be written as

(5) $$\mu \left(\Delta t\right)=\frac{\kappa {\left(\Delta t\right)}^{\kappa -1}}{\beta^{\kappa }},$$

where Δt is the elapsing time since the latest immigrant event, while κ and β are the shape and scale parameter of the Weibull distribution, respectively. In particular, κ=1 represents the standard Hawkes process with μ(t)=1/β. The algorithm proposed by Chen and Stindl was employed to estimate the parameters of the RHawkes process [17], which has the advantage of evaluating the likelihood function in quadratic time.

### 3.3. Goodness-of-Fit Tests

The goodness-of-fit tests on the Hawkes point process can be assessed through the residual analysis [29]. The residual $\xi_{i}$ can be calculated through integrating the estimated conditional intensity $\hat{\lambda }\left(t\right)$ from $t_{0}$ to $t_{i}$, such that

(6) $$\xi_{i}=\int_{t_{0}}^{t_{i}}\hat{\lambda }\left(t\right)dt$$

If the data are well calibrated by the Hawkes process, its residual process $\xi_{i}$ theoretically follows a Poisson distribution with λ=1, indicating that $\theta_{i}=\xi_{i}-\xi_{i-1}$ follows an independent identical exponential distribution with λ=1. Thus, the following two tests are performed on $\theta_{i}$ to check the quality of fits: (1) The Lagrange multiplier (LM) test is employed to test the autocorrelations. The null hypothesis is that there is no serial autocorrelation in residuals. We used 1–20 lags to check the existence of autocorrelations in $\theta_{i}$. The absence of autocorrelations ensures the independence of residuals. (2) The Kolmogorov–Smirnov (KS) test was adopted to check whether $\theta_{i}$ follows a standard exponential distribution. The null hypothesis is that the residual $\theta_{i}$ follows an exponential distribution with λ=1.

For the RHawkes process, we applied the method proposed by Chen and Stindl to conduct the goodness-of-fit tests [17]. Firstly, we mapped the estimated conditional intensity $\hat{\lambda }\left(t\right)$ to independent and uniformly distributed random variables $\left\{U_{n}\right\}$ in the interval [0,1] by the Rosenblatt transformation [30] such that

(7) $$U_{i}=1-\sum_{j=1}^{i-1}p_{ij}*\exp\left\{-\left[U\left(t_{i}-t_{j}\right)-U\left(t_{i-1}-t_{j}\right)\right]-\left[\Phi \left(t_{i}\right)-\Phi \left(t_{i-1}\right)\right]\right\},$$

where

(8) $$\left\{\begin{array}{ccc} p_{ij} & = & \left\{\begin{array}{cc} \frac{\phi (t_{i-1})}{\mu \left(t_{i-1}-t_{j}\right)+\phi \left(t_{i-1}\right)}\frac{d_{i-1,j}p_{i-1,j}}{\sum_{j=1}^{i-2}p_{i-1,j}d_{i-1,j}}, & j=1,\cdots ,i-2\\ 1-\sum_{k=1}^{i-2}p_{ik}, & j=i-1 \end{array}\right.\;,\\ d_{ij} & = & \left[\mu \left(t_{i}-t_{j}\right)+\phi \left(t_{i}\right)\right]\exp\left\{-\left[U\left(t_{i}-t_{j}\right)-U\left(t_{i-1}-t_{j}\right)\right]-\left[\Phi \left(t_{i}\right)-\Phi \left(t_{i-1}\right)\right]\right\}\\ U(t) & = & \int_{0}^{t}\mu (s)ds\\ \phi (t) & = & \sum_{j:t_{j}<t}\eta h(t-t_{j})\\ \Phi (t) & = & \int_{0}^{t}\phi (s)ds. \end{array}\right.$$

Please refer to Reference [17] for the detailed derivation of $\left\{U_{i}\right\}$. Secondly, we tested the independence and uniformity of $\left\{U_{i}\right\}$ through the LM test and KS test.

## 4. Results

As the crowdfunding donating activities exhibit a strong circadian rhythm, it is reasonable to calibrate the Hawkes and RHawkes processes for each day for each campaign. To ensure a reliable estimation, we excluded the days when the donating counts were less than 100 for each crowdfunding project, which resulted in 508,812 different windows. The donating events in these windows were further calibrated by the Hawkes and RHawkes processes. Before analyzing the calibrating results, the first step was to check whether the Hawkes and RHawkes processes can well-fit the daily donating events. The LM test and KS test were employed to assess the statistical significance of the calibrations. We counted the number of calibrations that passed the LM and KS tests, and both of them were at the significant levels of 1%, 5%, and 10% for each of the three Hawkes processes; the corresponding pass rates are presented in Table 1. Generally speaking, the three Hawkes processes, including the Hawkes process with the exponential memory kernel, the Hawkes process with the power-law memory kernel, and the renewal Hawkes process, all had a very good performance in describing the daily donating activities, as their pass rates of the LM tests and KS tests were greater than 91% at all significant levels. Furthermore, the pass rates of both tests were also greater than 90% at the level of 5%, indicating that the Hawkes processes fit the donating data very well. It can be found that the Hawkes process with the power-law memory kernel has the best fits since it always has the highest passing rate at the significant levels of 1%, 5%, and 10%. We also listed the Bayesian Information Criterion (BIC) values, which can be used to evaluate the goodness-of-fits, in Table 1. Again, we can see that the Hawkes process with the power-law memory kernel has the lowest average BIC value.

**Figure 2:**
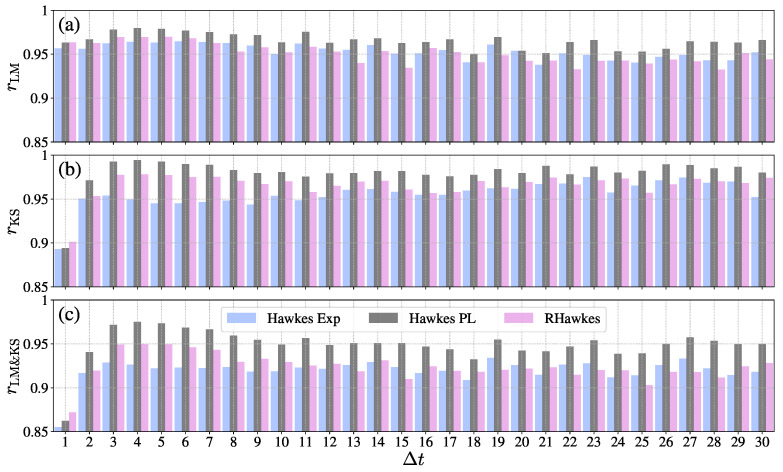
Plots of pass rates with respect to elapsing days Δt for different Hawkes processes. (**a**) Pass rates of LM tests. (**b**) Pass rates of KS tests. (**c**) Pass rates of both tests (LM and KS). The bar in each panel represents the fraction of calibrations passing the statistical tests at the significant level of 5%. In the analysis, we considered the elapsing days on which the crowdfunding campaign had more than 100 donations.

To further illustrate the goodness-of-fits of different Hawkes processes on different days, we plot the pass rates of the LM and KS tests at the significant level of 5% with respect to the elapsing days in Figure 2. One can observe that the pass rates of the three Hawkes processes are always above 90% on different days for LM tests, KS tests, and both tests (LM and KS). We found that the Hawkes model with the power-law memory kernel had a higher pass rate than the other two Hawkes processes, except on the creating day of crowdfunding projects Δt=1 when the RHawkes model fit the data best. A possible explanation is that the donating process on the first fundraising day is dominated by the bursts of immigrations (seed donors) and the RHawkes model has the advantage of accounting for the underlying correlations in immigrant events.

**Figure 3:**
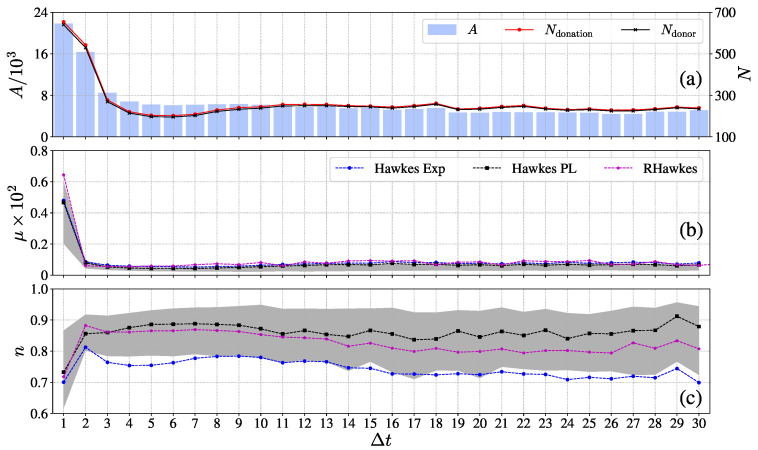
Plots of (**a**) fundraising amount *A*, number of donations $N_{\mathrm{donation}}$, and number of donors $N_{\mathrm{donor}}$; and (**b**) branching ratio *n* and (**c**) background intensity μ with respect to the elapsing days. The data point in each panel represents the average value estimated on that elapsing day. The shadow areas in panels (**b**,**c**) correspond to the 25–75% quantile range on that elapsing day for the Hawkes process with the power-law memory kernel. In the analysis, we considered the elapsing days on which the crowdfunding campaign had more than 100 donations.

**Figure 4:**
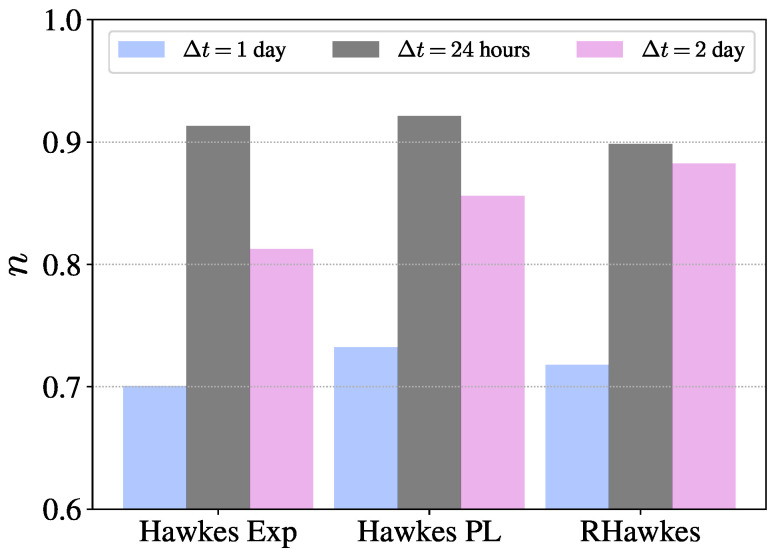
Bar plots of average branching ratio *n* on the first elapsing day (Δt=1 day), in the first elapsing 24 h (Δt=24 h), and on the second elapsing day (Δt=2 day) for the Hawkes process with the exponential memory kernel (Hawkes Exp), the Hawkes process with the power-law memory kernel (Hawkes PL), and the renewal Hawkes process (RHawkes), respectively. In the analysis, we considered the elapsing days on which the crowdfunding campaign had more than 100 donations.

The results of goodness-of-fit demonstrate the feasibility of uncovering the exogeneity and endogeneity in online donating activities with the Hawkes processes. Figure 3 plots the evolution of the average background intensity μ and average branching ratio *n* given by different Hawkes processes with respect to the elapsing days. For comparison, we also illustrate the evolving dynamics of donating characteristics, including the fundraising amount, the number of donations, and the number of donors. As shown in Figure 3a, one can observe that the donating characteristics of crowdfunding projects exhibit a dramatically decreasing pattern with the elapsing days and achieve a plateau after three days. The fundraising amount, the number of donations, and the number of donors in the plateau is about one-third of those on the first fundraising day. The evolution of the background intensity μ and the branching ratio *n* are plotted in Figure 3b,c for the three Hawkes processes. Each point represents the average value of the estimated background intensities and branching ratios on the corresponding elapsing days. The shadow area represents the 25–75% quantile range of μ and *n* for the Hawkes process with the power-law memory kernel. We can see that the background intensity exhibits a decreasing pattern and the branching ratio presents a rising trend. Both reach a plateau after three days. The decreasing and increasing pattern of μ and *n* can be explained as follows: (1) The first fundraising day usually cannot span over an entire day, resulting in a calibrating window of less than 24 h. As pointed out by Mark et al. [24], the branching ratio *n* can be underestimated in narrow windows. (2) The spreading process of the crowdfunding project is in an initial state on the first fundraising day. Thus, the probability of encountering seed donors (immigrant donating events) is relatively high, thus resulting in the decreasing pattern of background intensity [2]. Thus, we reestimated the branching ratio *n* of the donating events within the first 24 h after the crowdfunding project was created. The corresponding results are illustrated in Figure 4. For comparison, the branching ratios *n* on the first elapsing day and second elapsing day are also plotted. One can observe that the branching ratio in the first elapsing 24 h has the largest value. These results support the first explanation that narrow windows lower the estimation of the branching ratio and oppose the second explanation that the donating process in the first elapsing 24 h exhibits the strongest endogeneity, accounting for about 90% of the donations.

The estimated branching ratio *n* of the three Hawkes processes fluctuated in the range of 0.7–0.9, which indicates that the fundraising works in the sub-critical state. Our results also reveal that about 70–90% of online donations are endogenous, which is similar to the mid-price changes in financial markets [22,23,24].

## 5. Conclusions

In this paper, we aimed to quantify the fraction of online donations deriving from the endogeneity in crowdfunding campaigns, corresponding to endogenous feedback processes in which the donating and sharing actions of donors attract more people to donate. Following References [5,19,24], the level of endogeneity in online donations are measured by the branching ratio in the self-excited Hawkes process. By fitting three different Hawkes processes, including the Hawkes processes with an exponential memory kernel, the Hawkes processes with a power-law memory kernel, and the renewal Hawkes process, to the crowdfunding projects spanning over a period from October 2015 to June 2020, we found that more than 90% of the fits pass both LM and KS tests at the significant level of 5%. Our results reveal that the event data of online donations can be well-fitted by the Hawkes processes. We also found that the Hawkes model with the power-law memory kernel gives the best fits to the donating events, which is in accordance with the long-memory behavior in donating activities [15]. Furthermore, our results also provide evidence of the strong endogeneity in the online fundraising process, wherein about 70–90% of donations are triggered by the historical donations in crowdfunding campaigns. Even more interestingly, the average branching ratio *n* (index of endogeneity) is nearly a constant during the entire project period, even though the fundraising amount, number of donations, and number of donors shrink greatly with the elapsing days. In summary, our study presents a quantitative framework for disentangling the exogeneity and endogeneity in online charitable donations, which not only deepens our understanding of the online fundraising process but also expands the application of Hawkes processes in quantifying the exogenous and endogenous dynamics in complex systems.

## Figures and Tables

**Table 1:** Results of goodness-of-fits. This table lists the pass rates *r* of the LM and KS tests, and both of them were at the significant levels of 1%, 5%, and 10%. The average Bayesian Information Criterion (BIC) values (Ave. BIC) of all the fits are also listed for the Hawkes process with the exponential memory kernel (Hawkes Exp), the Hawkes process with the power-law memory kernel (Hawkes PL), and the renewal Hawkes process (RHawkes).

Model	α=1%			α=5%			α=10%			Ave. BIC
	$r_{\mathrm{LM}}$	$r_{\mathrm{KS}}$	$r_{\mathrm{LM}\&\mathrm{KS}}$	$r_{\mathrm{LM}}$	$r_{\mathrm{KS}}$	$r_{\mathrm{LM}\&\mathrm{KS}}$	$r_{\mathrm{LM}}$	$r_{\mathrm{KS}}$	$r_{\mathrm{LM}\&\mathrm{KS}}$	
Hawkes Exp	98.75%	95.73%	94.87%	95.86%	93.43%	90.27%	92.10%	91.35%	85.12%	4.621 $\times \;10^{3}$
Hawkes PL	99.43%	97.00%	96.53%	96.97%	95.70%	92.96%	93.45%	94.16%	88.26%	4.619 $\times \;10^{3}$
RHawkes	99.28%	96.81%	96.24%	96.44%	94.75%	91.63%	92.41%	92.38%	85.74%	3.237 $\times \;10^{18}$

## Data Availability

Publicly available datasets were analyzed in this study. This data can be found here: [https://www.qschou.com/], accessed on 19 November 2021.
